# Effect of Self-Myofascial Release of the Lower Back on Myofascial Gliding, Lumbar Flexibility, and Abdominal Trunk Muscle Strength: A Crossover Study

**DOI:** 10.3390/sports11080147

**Published:** 2023-08-02

**Authors:** Yuki Nakai, Katsutoshi Oe, Ryuko Matsuno, Ryoji Kiyama, Masayuki Kawada, Yasufumi Takeshita, Takasuke Miyazaki, Sota Araki

**Affiliations:** 1 Department of Mechanical Systems Engineering, Faculty of Engineering, Daiichi Institute of Technology, 1-10-2 Kokubuchuo, Kirishima City 899-4395, Japan; 2 Department of Mechanical and Electrical Engineering, Faculty of Engineering, Nippon Bunri University, 1727 Ichiki, Oita City 870-0397, Japan; ooekt@nbu.ac.jp; 3 Kirishima Orthopedics Clinic, 8-31 Kokubuchuoh, Kirishima City 899-4341, Japan; r_matsuno0728@yahoo.co.jp; 4 Department of Physical Therapy, School of Health Sciences, Faculty of Medicine, Kagoshima University, 8-35-1 Sakuragaoka, Kagoshima City 890-8544, Japan; kiyama@health.nop.kagoshima-u.ac.jp (R.K.); kawada@health.nop.kagoshima-u.ac.jp (M.K.); log.a.d.e.3.2.1@gmail.com (Y.T.); 5 Department of Orthopaedic Surgery, Graduate School of Medical and Dental Sciences, Kagoshima University, 8-35-1 Sakuragaoka, Kagoshima City 890-8544, Japan; takasuke0803@yahoo.co.jp; 6 Course of Physical Therapist, Department of Rehabilitation, Faculty of Health Sciences, Tohoku Fukushi University, 1-8-1 Kunimi Aoba-ku, Sendai City 981-8522, Japan; s-araki@tfu.ac.jp

**Keywords:** self-conditioning, core muscle strength, gliding, massage roller, echo

## Abstract

Roller massage has been recognized as an effective intervention for managing various conditions. However, data on the effects of roller massage on the dynamic mechanisms of the myofascial and soft tissues of the lower back are limited. This study aimed to examine the effect of the self-myofascial release of the lower back on myofascial gliding, lumbar flexibility, and abdominal trunk muscle strength using a roller massager. This crossover study included 24 college athletes who underwent three interventions—roller massage, static stretching, and control (rest). Before and after the intervention, lumbar and fascial gliding were evaluated using ultrasonography. Long-seat anteflexion (lumbar flexibility) and abdominal trunk muscle strength were assessed. The movement velocities of the subcutaneous tissue and the multifidus muscle over time were calculated using echo video analysis software, and gliding was estimated using the cross-correlation coefficient between the velocities. Gliding, lumbar flexibility, and abdominal trunk muscle strength showed significant intervention-by-time interactions. Roller massage significantly improved gliding, lumbar flexibility, and abdominal trunk muscle strength. The self-myofascial release of the lower back using a roller massager improved the lumbar/fascia gliding, lumbar flexibility, and abdominal trunk muscle strength compared to static stretching.

## 1. Introduction

Sports often have positive effects on health [1]; moreover, warming up and taking care of the body is important for athletes because they are often exposed to mechanical strain. Lumbar dysfunction in athletes can lead to withdrawal from training and competitions, poor performance, and a poor quality of life [2]. Thus, there is a need for an established method of caring for the lower back and maintaining trunk function in athletes. Myofascial release is often used for warm-ups and post-workout in sports [3]. Despite the popularity of myofascial release, its physiological effects are still being studied, and no consensus exists regarding the optimal program for a range of motion, recovery, and performance [4]. Myofascial release is performed to improve gliding between soft tissue layers, reduce pain, increase flexibility, and improve functional performance [5]. Mechanical stimulation has been previously reviewed for its biomechanical, physiological, neurological, and psychological effects [6]. A systematic review suggested that roller massage (RM) may be an intervention that increases joint range of motion without negatively impacting muscle performance [2]. However, most of these studies focused on the lower extremities. Data on the effects of RM on the dynamic mechanisms of myofascial and soft tissues in the lower back are limited. Currently, there are no reports on observed changes in gliding using echo video analysis. Although changes in gliding due to RM interventions can affect overall trunk function, the resulting changes in trunk muscle strength are unclear.

In contrast to myofascial release, static stretching (SS) has been the most common warm-up and care method used by athletes for several years [7]. This reduces the risk of injury by ensuring a sufficient joint range of motion for optimal exercise activity and muscle compliance [8]. A previous study that compared the effects of myofascial release to those of SS in the lower back showed that myofascial release improved trunk extensor strength, endurance, and range of motion more than SS [9]. Core stabilization exercises have been shown to improve core stability, endurance, and spinal mobility when combined with myofascial release with RM compared with core stabilization exercises alone [10]. The myofascial release of the lumbar region may affect the function of the deep trunk, stabilizing muscles attached to the spine via the thoracolumbar fascia; however, this is yet to be thoroughly investigated.

This study aimed to clarify the effects of self-myofascial release and SS interventions on the lower back, including the thoracolumbar fascia, using echo video analysis software and an abdominal trunk muscle strength measuring device. Previous studies have suggested that the greater the change in joint angle and the longer the moment arm of the muscle, the more the musculotendinous unit is stretched [11]. The moment arm of the lumbar multifidus muscle, the target of this study, is short during trunk movements [12]. This structural disadvantage makes it less effective in generating joint movements using the SS technique. Myofascial release increases the temperature of the skin, fascia, and muscle tissues owing to friction; furthermore, shear stress may be generated by applying direct and sweeping pressure. Thus, in college athletes, we hypothesized that RM would directly improve myofascial gliding, lumbar flexibility, and abdominal trunk muscle strength compared to SS. This study provides evidence of the dynamic mechanisms of both myofascial release and SS in the lumbar fascia and soft tissue. Athletes can easily perform self-myofascial release using RM. If self-myofascial release has a beneficial effect, this technique can be used as core conditioning to optimize lumbar and trunk functions. This may be useful for planning training programs, injury prevention strategies, or rehabilitation protocols, not only for athletes but also for older adults and patients requiring rehabilitation.

## 2. Materials and Methods

### 2.1. Participants

In this study, students who were members of a university indoor sports club (either basketball, volleyball, or futsal) were recruited by notification on a group social networking site, and those who were willing to participate were asked to volunteer. These students were chosen because they share a common involvement in sports that require running, jumping, a rapid change of directional movements, and high athleticism while moving around on an indoor court. A total of 24 college athletes (aged 20.1 ± 1.4 years; height 168.6 ± 8.1 cm; weight 62.5 ± 11.2 kg; competition history 8.8 ± 5.6 years), including six females, were enrolled. They exercised for 2–3 h, at least 2–4 times weekly. The exclusion criteria were as follows: the presence of low back pain, a history of neurological disease, a history of trunk and lumbar orthopedic disease, and surgery within 6 months before the initiation of this study. The sample size had a medium effect size (f = 0.25, α = 0.05, β = 0.80) after using G * Power 3.1.9 (Franz Faul, University of Kiel, Kiel, Germany) based on a previous study that analyzed the effect of SS on tissue stiffness [13]. After calculating the dropout rate, 24 participants were included.

### 2.2. Ethics

This study was approved by the Ethics Committee of the Daiichi Institute of Technology (21-002) and conducted in accordance with the Declaration of Helsinki. Written informed consent was obtained from all participants.

### 2.3. Procedures

**Figure 1:**
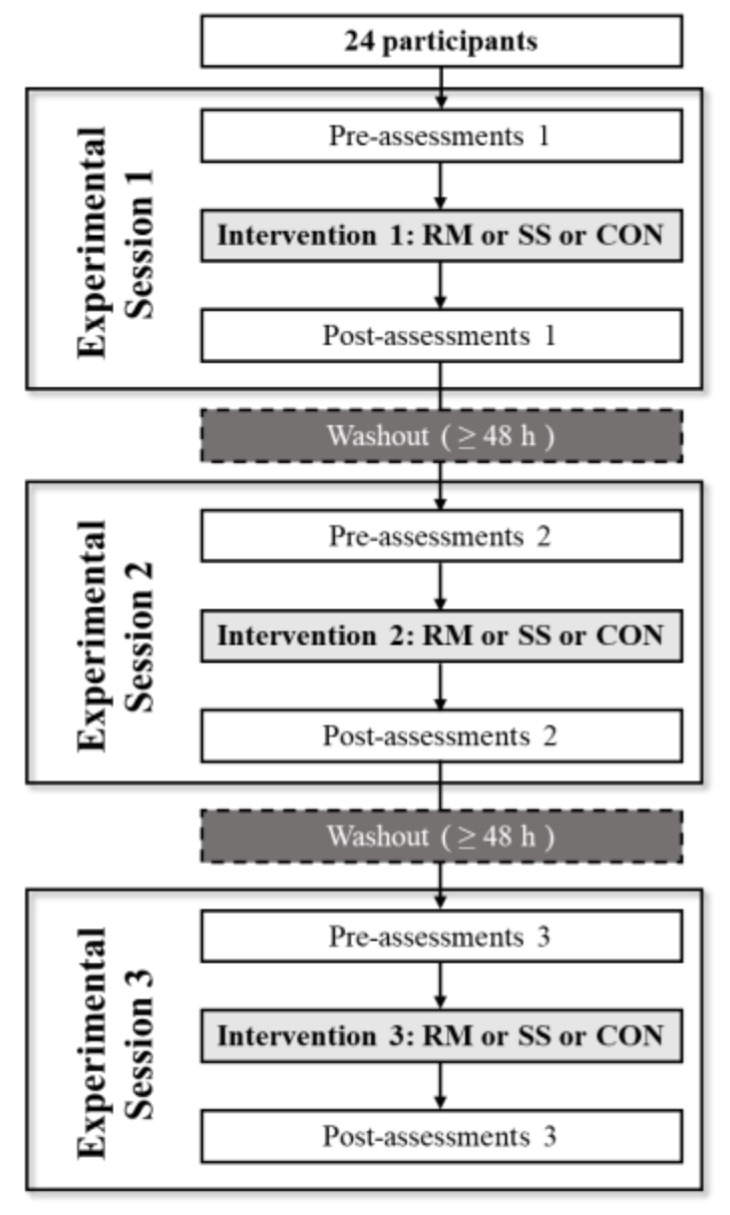
Flowchart of the crossover study: RM = roller massage; SS = static stretching; CON = control.

This was a crossover study in which three conditions (RM, SS, and control (CON)) were applied on three separate days (Figure 1). The participants were not divided into groups; all 24 participants were assessed for the RM, SS, and CON conditions on separate days. The order of the intervention was set randomly using a random number table with a washout period of ≥48 h [14]. The analytical indicators included lumbar fascial gliding, lumbar flexibility, and abdominal trunk muscle strength. The immediate effect was verified using pre- and post-assessments. The warm-up comprised 3 min of basic trunk movements: flexion, extension, lateral flexion, and rotation.

### 2.4. Intervention

**Figure 2:**
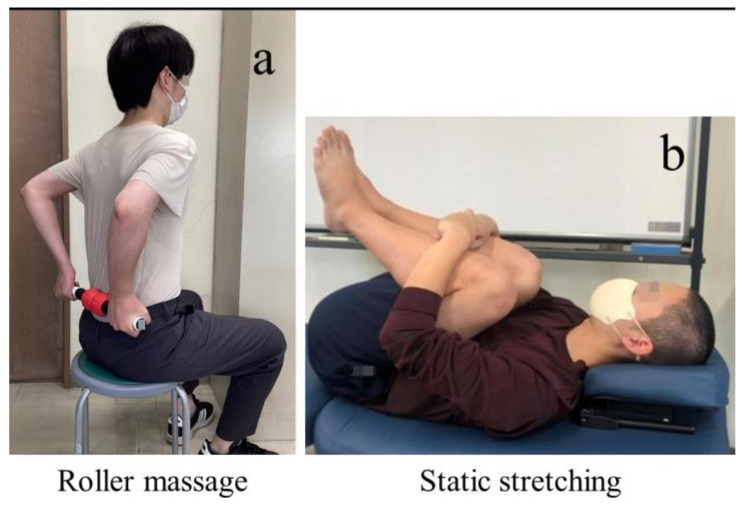
Intervention conditions: (**a**) roller massage; (**b**) static stretching.

A roller massager (7 × 45 × 7 cm, thermoplastic rubber + latex-free materials; Muscle Hand Roller, Balance1, Taiwan) was used for the RM intervention (Figure 2a). The roller massager was grasped with both hands in a sitting position on a 40 cm chair, and rolling was performed on the thoracolumbar fascia region, including the lumbar multifidus muscle (from L1 to the sacrum) [15]. The participants were instructed to hold their pelvic spine in a neutral position. The rolling on the target region was repeated for 2 s using a metronome (ME-150, Yamaha, Shizuoka, Japan) set at 60 bpm. The movement involved going up and down in the target region for 2 s each time [14]. The intervention included three 30 s sessions for a total of 90 s, with an interval of 30 s [16]. The pressure was set at 7/10 using a numerical rating scale (0 = no discomfort and 10 = maximum discomfort) [17]. SS was performed on the trunk extensor muscles, including the lumbar multifidus muscle, in the supine position with the knees held with both hands (Figure 2b) [9]. To minimize the potential effect of stretching on muscle strength, each participant remained in the final position for 30 s while maintaining a stretch intensity of 7/10 on a numerical rating scale [18]. The total intervention time was the same as that of the RM, with three sets of 30 s each and an interval of 30 s. Participants in the CON group remained seated on a chair for 2 min and 30 s.

### 2.5. Measurement Method of Lumbar Myofascial Gliding

**Figure 3:**
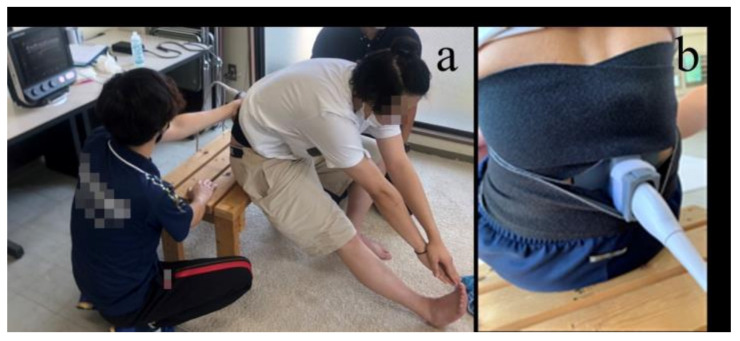
The movement of the myofascial membrane of the lower back is imaged with an ultrasonic diagnostic imaging device. (**a**) The movement of the lower back when the trunk bends forward and returns is measured; (**b**) the probe is attached to the low back with a fixation device created with a 3D printer.

Myofascial gliding in the lumbar region was evaluated using an ultrasonic diagnostic imaging device (SonImage MX1; KONICA MINOLTA, Tokyo, Japan). The participants sat on a 40 cm chair, extended one leg forward in a pelvic neutral position, bent forward to reach their toes, and then returned to the starting position (Figure 3a). Using the metronome, the participants bent forward for 3 s, held the position for 2 s, and then returned to the starting position in 3 s; thus, a total of 8 s of motion were repeated three times. The measurements were performed after completing the timing practice.

Ultrasound motion images were captured in B-mode using a high-sensitivity wideband linear probe (L11-3; KONICA MINOLTA, Tokyo, Japan). The target area was set to 2 cm outside the spinous process of L4. The long axis was imaged [19], and the sampling frequency was set at 30 Hz. To prevent the probe from moving during the intervention, a probe fixation device was drawn using a computer-aided design (Rhino7, Robert McNeel & Associates, Seattle, WA, USA) and created using a 3D printer (Form2, Formlabs, MA, USA) (Figure 3b). In this study, one experienced physical therapist performed the ultrasound echo manipulation and analysis. The measurement environment and methods were tested in advance.

**Figure 4:**
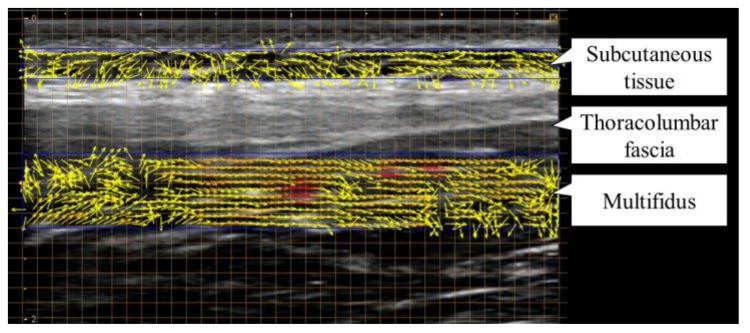
Echo video analysis software quantifying the rate of movement of the subcutaneous tissue and multifidus muscles on echo images.

Echo video analysis software (Echolizer, GLAB, Hiroshima, Japan) was used to quantify the movement velocity of the muscles and other soft tissues in the echo images (Figure 4). This software uses the Farneback method (frame offset: 3), which calculates the optical flow by layering images [20]. The validity and reliability of the Echolizer software in quantifying tissue motion using ultrasound images have been previously reported [21]. Excellent accuracy has been demonstrated with a relative error of 0.2% after applying regression equations within the program. The location and size of the region of interest (ROI) were defined using a grid that overlaid the ultrasound images for reproduction. In the ROIs, the X-axis was defined as head–lower limbs, and the Y-axis was defined as abdomen–back. The ROIs were set with the thoracolumbar fascia as the boundary at the following two locations: the superficial region, which was the subcutaneous tissue (x-axis, 36 mm × y-axis, 2 mm), and the deep region, which was the multifidus muscle (x-axis, 36 mm × y-axis, 5 mm). The average velocity along each X-axis (head–lower limb direction) was then calculated. The cross-correlation coefficient between the time series of the subcutaneous tissue and the multifidus muscle movement velocities was calculated, and the average of three trials was adopted as the representative value. A high cross-correlation coefficient indicated that the two tissues moved similarly and gliding was reduced, whereas a low cross-correlation coefficient indicated that the two tissues moved independently and gliding increased. These indications were based on a previous study investigating both the vastus lateralis and subcutaneous tissue gliding during knee movement [22].

### 2.6. Measurement Method of Flexibility

The sit-and-reach test was performed using a long-seat anteflexion measuring instrument (SH; TOEI I LIGHT, Saitama, Japan) to evaluate the flexibility of the lower back muscles and fascia. The measurement was performed in the limb position with both feet separated by approximately 25–30 cm [23]. During the evaluation, the participants placed their fingertips on top of each other, touched the yardstick, extended their arms, dropped their heads, slowly pushed forward, and held the position for 2 s. The same examiner confirmed that the participant’s knees remained extended. After one or two practice trials with minimal movements to obtain a better understanding of the measurement behavior, three measurements were taken, and the mean value was calculated.

### 2.7. Measurement Method of Abdominal Trunk Muscle Strength

**Figure 5:**
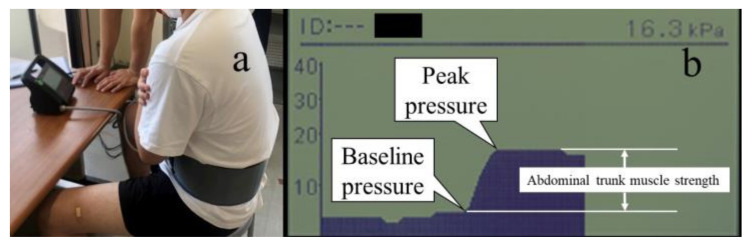
Abdominal trunk muscle strength measurement: (**a**) measured by wrapping the cuff around the abdomen; (**b**) the difference between the peak pressure and the baseline pressure is calculated.

Abdominal trunk muscle strength was measured (Figure 5a) using an abdominal trunk muscle strength-measuring device (RECORE, Sigmax, Tokyo, Japan). The measurements were taken isometrically and statically in the sitting position, with both upper limbs crossing in front of the chest at the pelvic–spinal midline. After wrapping a cuff around the abdomen and applying an approximate pressure of 5 kPa to the abdominal wall (baseline pressure), the participants were instructed to maximize the abdominal pressure (peak pressure), and the amount of change in pressure (peak pressure–baseline pressure) was then measured as the abdominal trunk muscle strength (Figure 5b). A previous study reported that this abdominal trunk muscle strength-measuring device promoted muscle activity in the diaphragm, rectus abdominis, internal and external abdominal oblique, transversus abdominis, and pelvic floor muscles [24]. After no fewer than two practice trials with minimal force to better understand how to apply force, three measurements were taken, and the mean value was calculated.

### 2.8. Statistical Analysis

Data for each item of the RM, SS, and CON are presented as means and standard deviations. The data distribution of each item was determined to follow a normal distribution using the Shapiro–Wilk test. To examine the effects of repeated measures with pre- and post-intervention as one factor and the three conditions (RM, SS, and CON) as another factor, we conducted a two-way analysis of variance for gliding, lumbar flexibility, and abdominal trunk muscle strength. The sphericity test was based on Mauchly’s test, and the Greenhouse–Geisser correction was used if the sphericity was significant. When an interaction was observed, post hoc comparisons were made using a paired *t*-test with pre- and post-intervention values for each condition (RM, SS, CON). A change in the value obtained by subtracting the value before the intervention from the value after the intervention was considered positive. The reliability of the ultrasonic echo gliding coefficient, lumbar flexibility, and abdominal trunk muscle strength was analyzed using the intraclass correlation coefficient (ICC_1,3_) and was measured three times. Statistical analysis software (SPSS version 27.0, IBM, NY, USA) was used for processing, and the significance level was set at *p* < 0.05. The effect size (d), calculated for comparison before and after each intervention, corresponded to the following criteria: trivial, <0.2000; small, 0.2000–0.5000; medium, 0.5000–0.8000; and large, >0.8000 [25]. The effect size (partial *η*^2^) calculated for the analysis of variance was classified according to the following criteria: trivial, <0.0099; small, 0.0099–0.0588; medium, 0.0588–0.1379; and large, >0.1379 [26].

## 3. Results

Table 1 presents the intervention results for the three conditions. The gliding coefficient showed a significant interaction (F = 5.358, *p* = 0.008, partial *η^2^* = 0.189: large), and a post hoc comparison showed that the cross-correlation coefficient decreased only with RM and improved gliding (−0.080, *p* = 0.004, *d* = 0.66: medium). Lumbar flexibility showed a significant interaction (F = 5.135, *p* = 0.010, partial *η^2^* = 0.183: large), and the post hoc comparison showed improved lumbar flexibility only with RM (+1.27 cm, *p* = 0.001, *d* = 0.77: medium). Abdominal trunk muscle strength also showed significant interactions (F = 6.081, *p* = 0.005, partial *η^2^* = 0.209: large), and post hoc comparisons showed improvement in abdominal trunk muscle strength only with the RM (+1.56 kPa, *p* < 0.001, *d* = 0.85: large). The ICC _(1,3)_ of the cross-correlation coefficient between the subcutaneous tissue and the multifidus muscle, which indicates gliding, was 0.828–0.941 (95% confidence interval = 0.663–0.972, *p* < 0.001), indicating significant reproducibility. The ICCs _(1,3)_ of lumbar flexibility and abdominal trunk muscle strength measured three times were 0.995–0.998 (95% confidence interval = 0.991–0.999, *p* < 0.001) and 0.975–0.983 (95% confidence interval = 0.952–0.992, *p* < 0.001), respectively. Furthermore, both showed high reliability.

## 4. Discussion

This study examined the effects of the self-myofascial release of the lower back muscles, including the thoracolumbar fascia, on changes in muscle/fascia dynamics, lumbar flexibility, and abdominal trunk muscle strength. Only the RM intervention improved gliding, lumbar flexibility, and abdominal trunk muscle strength.

In previous studies that used ultrasonic echo video analysis software, improved gliding between the subcutaneous tissue and the vastus lateralis muscle after a greater trochanteric fracture was associated with improved lateral thigh pain [22]. The effect of a decrease in the cross-correlation coefficient between subcutaneous tissue and muscle was similar to that observed in our study. The deep layers of subcutaneous adipose tissue form a mobile layer that facilitates smooth movement in the musculoskeletal system and plays an important role in inter-tissue gliding [27]. Hyaluronic acid has been suggested to play a role in fascia-to-muscle gliding [28]. As the shear rate increases, the hyaluronic acid molecules do not quickly return to their undisturbed shape and become less viscous [29]. This study showed significant improvement in gliding with RM intervention. The constant rate of pressure with RM may have increased fluid pressure, tissue temperature, and gliding; reduced viscosity between tissues; and improved gliding between myofascial layers.

Only the RM intervention showed a significant increase in lumbar flexibility. This result is consistent with recent evidence of the positive effects of RM interventions [14]. Fascia release is a method of direct pressure application that reduces fascia densification and strain and has been mechanically and neurophysiologically proven to induce fascial fiber changes, cellular responses, piezoelectric effects, and blood flow [5]. Previous studies have also reported that RM interventions alter spinal excitability and affect the range of motion [30]. Lumbar dysfunction is characterized by deep trunk weakness and overactivity of superficial muscles, including the erector spinae muscles [31,32]. In contrast, myofascial release directly acts on muscles and soft tissues and decreases the resting activity of the paraspinal muscles of the lumbosacral spine [33]. In our study, RM interventions were performed on the lower back, which may have directly improved lumbar flexibility. However, no significant improvement in lumbar flexibility was observed with the SS intervention. The multifidus muscle, located in the lumbar region of the trunk, causes trunk extension, ipsilateral lateral flexion, and contralateral rotation; however, the movement of the lumbar facet joint during exercise is <6° [34]. Stretching can be difficult because of the relationship between the origin and insertion of the multifidus muscle and muscle length [35].

RM intervention in the lumbar region improved abdominal trunk muscle strength. Previous studies have reported that reduced stiffness of the thoracolumbar fascia caused by fascia release and subsequent relaxation allows the trunk extensors to function more efficiently [9]. A synergistic effect was observed between the multifidus and transversus abdominis muscles. Those who performed isometric contraction of the multifidus muscle showed 4.5 times higher transversus abdominis muscle contraction than those who performed poorly, and poor multifidus muscle contraction correlated with poor transversus abdominis muscle contraction [36]. The trunk-stabilizing muscles, including the internal oblique, transversus abdominis, and multifidus muscles, are attached to the spine via the thoracolumbar fascia and contribute to trunk stability [37]. These myofascial muscles work together to create a corset-like system with balanced tension [38]. Synergistic effects between the core muscles have been reported in systematic reviews [39]. Increased thickness during the transversus abdominis muscle contraction has been shown in healthy participants after an invasive intervention on the lumbar multifidus muscle [40]. Results from cadaveric and animal studies suggest that structural and molecular changes in myofascia affect force transmission during exercise [41]. Previous studies have shown that massage increases skin and intramuscular temperatures [42], which increases muscle output [43,44]. The RM intervention in this study may have affected the force transfer of the abdominal trunk muscles owing to the myofascial coupling of the core musculature via the multifidus muscles. In our study, muscle strength did not improve after SS intervention, which is consistent with the results of a previous study [9]. No decrease in muscle strength was observed, possibly because effective stretching is difficult to perform. Therefore, routine self-myofascial release with RM may provide proper care for myofascial structures and contribute to performance improvements and injury prevention in college athletes.

This study had some limitations. First, it only included young college athletes without low back pain; therefore, it is unclear whether the results apply to other age groups or people with low back pain. Second, we did not assess the changes in body temperature or the autonomic nervous system. Third, the participants were subjected to subjective intensity in the RM and SS interventions; therefore, individual differences in intensity are a potential limitation. Fourth, the myofascial gliding measurements and sit-and-reach tests included movements in which the hamstrings were extended, which could have affected the results of this study, although there was no direct intervention on the hamstrings. Fifth, due to the absence of blinding among participants and researchers, the possibility of introducing bias or influencing the results cannot be ruled out. Sixth, the same procedures were used for each intervention, with appropriate warm-up exercises, and the participants were encouraged to give their maximum effort each time. Nevertheless, the participant expectations and the warm-up exercises may have had an impact on the results. Finally, although the immediate effects were investigated, the long-term effects were not; therefore, future research is needed.

## 5. Conclusions

The results of this study suggest that the RM intervention immediately improves myofascial gliding, lumbar flexibility, and abdominal trunk muscle strength. RM may be useful for the self-myofascial release of the lumbar back. We recommend that college athletes incorporate this technique into their daily core conditioning routine to optimize lumbar and trunk functions. This may contribute to the prevention of back organic disorders and physical performance. RM may also be helpful in planning training programs and rehabilitation protocols for individuals with low back pain due to myofascial issues and those who wish to improve it.

## Figures and Tables

**Table 1:** Comparison of each condition before and after intervention.

		Pre-Intervention	Post-Intervention	Change Value [95% CI]	ES	Interaction		ES
					d	F	P	Partial η^2^
**Gliding**	**RM**	0.73 ± 0.13	0.65 ± 0.15	−0.08 ± 0.12 ** [−0.13, −0.03]	0.66	5.358	0.008 **	0.189
	**SS**	0.71 ± 0.11	0.70 ± 0.11	−0.01 ± 0.12 [−0.06, 0.04]	0.08			
	**CON**	0.70 ± 0.13	0.72 ± 0.13	0.01 ± 0.09 [−0.03, 0.05]	0.15			
**Lumber flexibility (cm)**	**RM**	37.0 ± 10.6	38.3 ± 10.1	1.27 ± 1.65 ** [0.57, 1.96]	0.77	5.135	0.010 *	0.183
	**SS**	37.8 ± 10.4	38.5 ± 10.3	0.67 ± 1.70 [−0.05, 1.38]	0.39			
	**CON**	37.6 ± 10.3	37.3 ± 10.0	−0.23 ± 1.31 [−0.78, 0.32]	0.18			
**Abdominal trunk muscle strength (kPa)**	**RM**	20.4 ± 6.2	22.0 ± 6.2	1.56 ± 1.82 ** [0.79, 2.33]	0.85	6.081	0.005 **	0.209
	**SS**	21.6 ± 6.3	22.1 ± 6.0	0.54 ± 1.56 [−0.12, 1.20]	0.34			
	**CON**	21.5 ± 5.6	21.2 ± 5.9	−0.24 ± 1.92 [−1.05, 0.57]	0.13			

Mean ± standard deviation, 95% CI = 95% confidence interval, ES = effect size, RM = roller massage, SS = static stretching, CON = control, ** *p* < 0.01, * *p* < 0.05 at post hoc test.

## Data Availability

The datasets generated and/or analyzed in the current study are available from the corresponding authors upon reasonable request.
